# Biogeosciences Perspectives on Integrated, Coordinated, Open, Networked (ICON) Science

**DOI:** 10.1029/2021EA002119

**Published:** 2022-03-24

**Authors:** D. Dwivedi, A. L. D. Santos, M. A. Barnard, T. M. Crimmins, A. Malhotra, K. A. Rod, K. S. Aho, S. M. Bell, B. Bomfim, F. Q. Brearley, H. Cadillo‐Quiroz, J. Chen, C. M. Gough, E. B. Graham, C. R. Hakkenberg, L. Haygood, G. Koren, E. A. Lilleskov, L. K. Meredith, S. Naeher, Z. L. Nickerson, O. Pourret, H.‐S. Song, M. Stahl, N. Taş, R. Vargas, S. Weintraub‐Leff

**Affiliations:** ^1^ Earth and Environmental Sciences Area Lawrence Berkeley National Laboratory Berkeley CA USA; ^2^ Department of Environmental Engineering Federal University of Paraná Polytechnic Center Campus Curitiba Brazil; ^3^ Institute of Marine Sciences University of North Carolina at Chapel Hill Morehead City NC USA; ^4^ School of Natural Resources and the Environment USA National Phenology Network University of Arizona Tucson AZ USA; ^5^ Department of Earth System Science Stanford University Stanford CA USA; ^6^ Earth and Biological Sciences Directorate Pacific Northwest National Laboratory Richland WA USA; ^7^ National Ecological Observatory Network Battelle Boulder CO USA; ^8^ Institute of Environmental Science and Technology (ICTA) Universitat Autònoma de Barcelona (UAB) Bellaterra Spain; ^9^ Climate and Ecosystems Sciences Division Lawrence Berkeley National Laboratory Berkeley CA USA; ^10^ Department of Natural Sciences Manchester Metropolitan University Manchester UK; ^11^ School of Life Sciences Arizona State University Tempe AZ USA; ^12^ Department of Geography, Environment, and Spatial Sciences Michigan State University East Lansing MI USA; ^13^ Department of Biology Virginia Commonwealth University Richmond VA USA; ^14^ School of Biological Sciences Washington State University Richland WA USA; ^15^ School of Informatics, Computing & Cyber Systems Northern Arizona University Flagstaff AZ USA; ^16^ Department of Geosciences The University of Tulsa Tulsa OK USA; ^17^ Boone Pickens School of Geology Oklahoma State University Stillwater OK USA; ^18^ Copernicus Institute of Sustainable Development Utrecht University Utrecht The Netherlands; ^19^ Northern Research Station USDA Forest Service Houghton MI USA; ^20^ School of Natural Resources and the Environment University of Arizona Tucson AZ USA; ^21^ Department of Surface Geosciences GNS Science Lower Hutt New Zealand; ^22^ AGHYLE UniLaSalle Beauvais France; ^23^ Department of Biological Systems Engineering University of Nebraska–Lincoln Lincoln NE USA; ^24^ Department of Food Science and Technology University of Nebraska–Lincoln Lincoln NE USA; ^25^ Department of Geosciences Union College Schenectady NY USA; ^26^ Department of Plant and Soil Sciences University of Delaware Newark DE USA

**Keywords:** Integrated, Coordinated, Open, Networked, community, citizen

## Abstract

This article is composed of three independent commentaries about the state of **I**ntegrated, **C**oordinated, **O**pen, **N**etworked (ICON) principles in the American Geophysical Union Biogeosciences section, and discussion on the opportunities and challenges of adopting them. Each commentary focuses on a different topic: (a) Global collaboration, technology transfer, and application (Section 2), (b) Community engagement, community science, education, and stakeholder involvement (Section 3), and (c) Field, experimental, remote sensing, and real‐time data research and application (Section 4). We discuss needs and strategies for implementing ICON and outline short‐ and long‐term goals. The inclusion of global data and international community engagement are key to tackling grand challenges in biogeosciences. Although recent technological advances and growing open‐access information across the world have enabled global collaborations to some extent, several barriers, ranging from technical to organizational to cultural, have remained in advancing interoperability and tangible scientific progress in biogeosciences. Overcoming these hurdles is necessary to address pressing large‐scale research questions and applications in the biogeosciences, where ICON principles are essential. Here, we list several opportunities for ICON, including coordinated experimentation and field observations across global sites, that are ripe for implementation in biogeosciences as a means to scientific advancements and social progress.

## Key Points

- Biogeosciences needs Integrated, Coordinated, Open, Networked (ICON) principles to address multiscale global problems and reduce geographical bias in scientific progress
- Much potential exists for emphasizing people‐centric capacity building, involving relevant stakeholders within an ICON framework
- Globally coordinated experimental and field data provide challenges and opportunities for scientific advancement in biogeosciences

## 1 Introduction

Integrated, Coordinated, Open, Networked (ICON) science aims to enhance synthesis, increase resource efficiency, and create transferable knowledge (Goldman et al., 2021). In particular, ICON science is an approach to designing and carrying out research activities encompassing four components:

1. Integrating processes across traditional disciplines
2. Coordinating consistent protocols across systems to generate interoperable data across systems
3. Openly exchanging ideas, data, software, and models, and
4. Promoting networks and collaborations that benefit and provide resources toward common scientific goals synergistically

Biogeosciences is an inherently interdisciplinary field that needs ICON to address grand environmental challenges, including anthropogenic climate change and its effects on abiotic and biotic systems. Tackling multiscale global problems requires reducing geographical bias in data collection and scientific progress. Integrating biology, chemistry, and Earth sciences, the biogeosciences address human impacts on the biophysical and chemical properties of terrestrial and aquatic systems around the globe. However, a variety of hurdles prevent ICON implementation in biogeosciences. As part of a collection of commentaries spanning ICON in the geosciences (Goldman et al., 2021), this article evaluates the state of ICON in biogeosciences and focuses on three aspects surrounding global collaborations, stakeholder engagement, and data research and application in biogeosciences.

## 2 Global Collaboration, Technology Transfer, and Application

### 2.1 The Need for ICON in Biogeosciences

**Figure 1:**
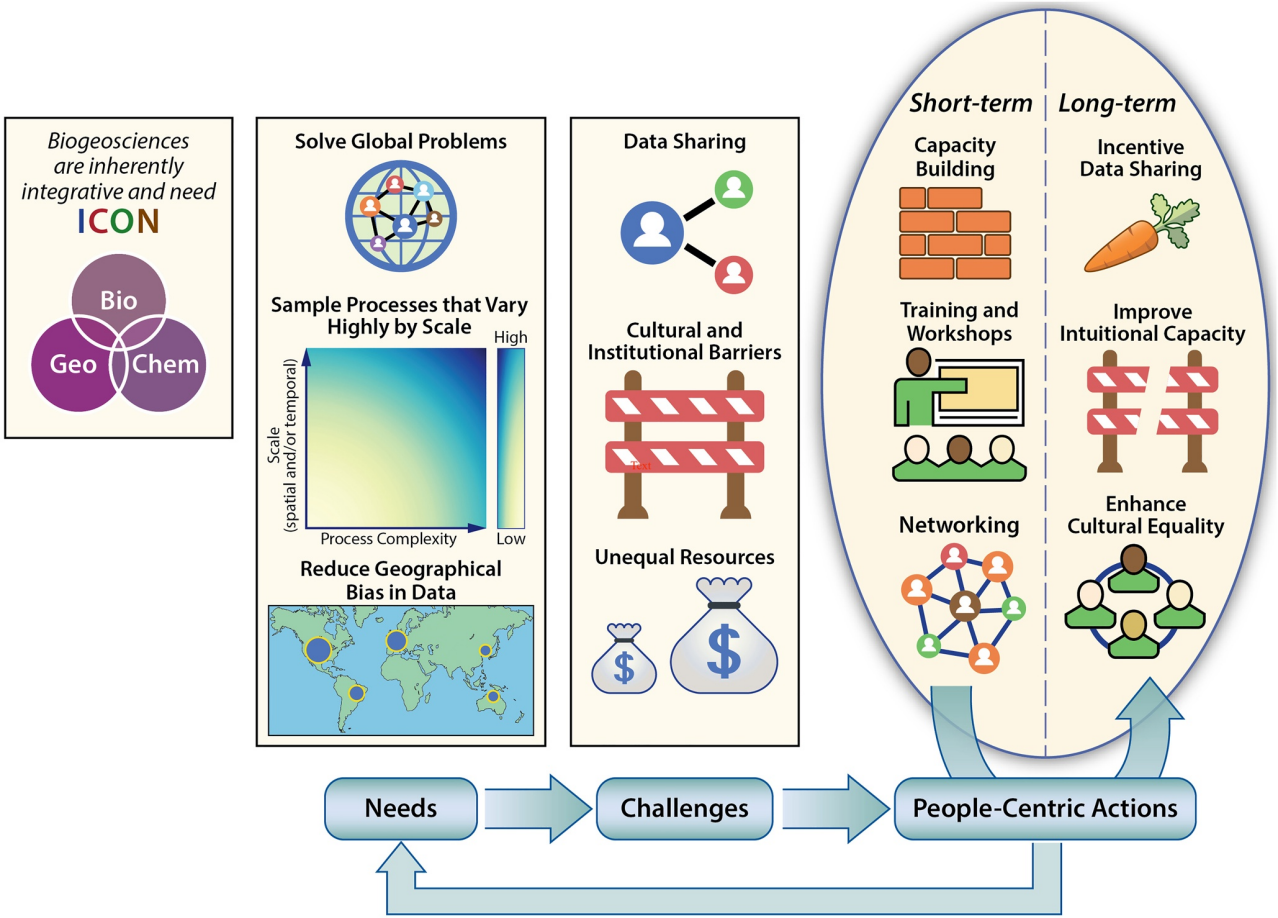
Biogeosciences, an inherently interdisciplinary field, **needs** ICON to address urgent and multiscale global problems, where process‐complexity and need for ICON increase with scale, and to reduce geographical bias in data and scientific progress. Various **challenges** hinder ICON in biogeosciences, but perhaps the most critical ones revolve around cultural and institutional barriers that prevent data sharing and cross‐border collaborations. Our recommended **short‐ and long‐term** solutions focus on **people‐centric actions** to break these barriers, especially for low‐to‐middle‐income countries (LMIC) researchers.

Many pressing grand environmental challenges, including climate change and nutrient deposition, are global in scope and transcend political boundaries (Figure 1). These challenges are linked to local‐to‐global ecosystem processes (e.g., carbon or nitrogen cycling) that require distributed observations across spatial scales. Too often, measurements and networks are defined within political boundaries and concentrated in high‐income countries, leading to geographical biases (e.g., Stell et al., 2021). Appropriately addressing these grand challenges requires research to be conducted across countries in a coordinated way. However, participation costs can be prohibitive, especially for developing countries. Given this barrier and the heterogeneity of methods available in biogeosciences, we must develop strong instrumentation and protocol coordination for characterizing biogeochemical pools and fluxes, data archiving, and researcher training (e.g., Hubbard et al., 2018, 2020; Varadharajan et al., 2019). Overall, tackling biogeosciences grand challenges requires concerted actions that are **integrated, coordinated, open, and networked**. Below, we briefly describe several hurdles toward implementing the ICON principles and discuss the path forward for pioneering global biogeosciences.

### 2.2 Major Challenges

Grand challenges in the field of biogeosciences are global and require international collaborations to address them. **Integrated and coordinated** efforts are needed for success, but organizational and cultural challenges for global collaborations present barriers to interoperability (Villarreal & Vargas, 2021). Organizational barriers relate to challenges regarding institutional responsibility and authority, as well as the inequality of resources (Mirtl et al., 2018; Vargas et al., 2017). Cultural barriers relate to how scientists perceive the world and their relationships and collaborations. Differences in cultural norms, institutions, education, socioeconomic status, modes of communication, language, infrastructure, and technology complicate collaboration between scholars from different regions, institutes, or subdisciplines. Recent decades have witnessed an enthusiasm for collaborating in education and research, as most scholars recognize the importance of joint efforts in seeking solutions for global environmental issues. Therefore, **open and networked** efforts are also needed to address grand challenges in the field of biogeosciences. However, cultural barriers can hinder networking, and institutions may prioritize perceived national over international interests. Even within national borders, there are barriers to open data sharing, such as the desire to avoid competition between smaller and larger research groups due to the availability of disparate resources. International cultural and resource differences further intensify barriers to open and networked efforts, because scholars from developing countries may not receive equal recognition for the outcomes of their data (e.g., inclusion on publications, patents) (Armenteras, 2021). This recognition is critical for addressing local issues, maintaining rigorous research and education programs for their groups, and career and institutional advancement.

### 2.3 Looking Forward: An Urgent Call for People‐Centric Actions

To address barriers to ICON in the biogeosciences, we call for people‐centric actions. In the short term, investment in capacity‐ and infrastructure‐building, workshops, and training can help overcome barriers to global collaboration. These efforts will favor the development of researchers with a sense of “belonging” to global networks, and will facilitate reducing technological barriers (e.g., infrastructure) and global cooperation. Scientific societies, institutions of higher learning, and other research entities can promote these coordinated efforts by organizing in‐person and virtual events. For longer‐term actions, we propose top‐down incentives that reward ICON activities, such as data sharing (e.g., publishing open datasets) and efforts towards integration and coordination of networked efforts (e.g., activities that support or develop networks such as LTER, FLUXNET, ForestPlots). Further, recognizing the need for close coordination and integration across the globe to advance science, the Accelerating Research through International Network‐to‐Network Collaborations (AccelNet) program of the National Science Foundation (NSF) fosters connections among research networks of the United States of America (USA) and complementary networks abroad. As an illustration, Arora et al. (2021) organized several workshops supported by the NSF and Department of Energy (DOE) between 2019 and 2021 and brought together an international network of DOE watersheds and critical zone observatories (CZOs). They emphasized that networks can serve as a vehicle for knowledge exchange, integration, and discovery among researchers of the USA and their international counterparts. These efforts should carry as much weight as scientific publications for hiring and promotions. Furthermore, longer‐term funding priorities are needed for institutionalizing capacity and reducing entry costs, especially for researchers from low‐to‐middle income countries (Figure 1). Overall, biogeosciences deal with cross‐scale and cross‐continental problems, and need ICON principles.

## 3 Community Engagement, Community Science Education, and Stakeholder Involvement

### 3.1 Current State of ICON

In recent years, increased engagement among non‐scientists through public participation in the scientific endeavor (Besançon et al., 2021; Roy et al., 2012) has significantly boosted and popularized community or citizen science projects. These projects are supported by volunteers and have the potential to yield consistently collected diverse data (**integrated and coordinated**) that are openly accessible (**open**) through stakeholder engagement (**networked**). In this commentary, although we use “community science” as an umbrella term for “citizen science,” “public participation with science,” and “advancing science through volunteer‐contributed data,” these terms may have slightly varying connotations in different scientific fields. As an illustration of community science projects, the USA‐based phenology‐focused community science program Nature's Notebook utilizes the same published and scientifically vetted observation protocols as the National Ecological Observation Network (NEON; Denny et al., 2014; Elmendorf et al., 2016), and provides ready access to data contributed by both community and professional (e.g., NEON) scientists (**coordinated, open**). Similarly, many research projects taking place at International Long‐Term Ecological Network sites engage students in **integrated** and **coordinated** research (Gosz et al., 2010).

However, examples of coordinated, open, networked science engaging communities, stakeholders, and community scientists remain rare. Funding for science and scientific publishing are two areas where changed practices are leading to increased engagement among communities, stakeholders, and community scientists. In Australia, New Zealand, Japan, and several European countries, publicly funded research projects are required to involve local stakeholders and indigenous communities. Federally funded research proposals in New Zealand must demonstrate direct involvement and/or benefit to Māori and address indigenous knowledge and innovation, societal and health concerns, and environmental sustainability (Ministry of Research, Science & Technology, 2005). In the USA, expectations for outreach are variable: the DOE calls require outreach plans, and the NSF encourages but does not require outreach and education through grant‐funded “broader impacts”. In addition to federal agencies, several non‐federal, state‐level, and university‐based programs also require stakeholder engagement (e.g., SeaGrant Programs, Water Resources Institutes). As a cultural change, a growing number of journals are innovating by ensuring the entire peer‐review process is transparent, accessible, and available for an open viewing and comment by not only scientists, but also stakeholders and policymakers.

### 3.2 Major Challenges

A key challenge to engaging stakeholders, community members, and non‐professional scientists in ICON science is the limited awareness of or access to **coordinated** established and often technical research protocols, **open data efforts**, and communication channels used by professional scientists. Monitoring protocols are not always readily available for would‐be non‐professional data collectors or users. Similarly, data repositories and communication channels used by professional scientists remain relatively unknown or inaccessible to stakeholders, community members, and non‐professional scientists, challenging efforts to engage these communities while adhering to ICON principles. Such limited ICON‐centered interaction between scientists and non‐scientists stifles the transfer, application, and translation of global change research that could shape policy and inform decision‐making (Enquist et al., 2017). Another important barrier to greater engagement among scientists and researchers with stakeholders and community members is the persistent and intensifying academic standard that productivity and impact be judged primarily via peer‐reviewed articles (Davies et al., 2021; Perkmann et al., 2021). This results in many research findings and data remaining “untranslated” for a non‐technical audience, rendering potentially valuable results inaccessible to policymakers, stakeholders, and the general public. Further, scientific journals and databases are frequently inaccessible to the public, presenting additional barriers to open, coordinated science engaging and used by stakeholders, community members, or community scientists.

### 3.3 Opportunities to Advance Biogeosciences Through Community Science

The intentional engagement of local stakeholders, community members, and educators during the development of a research project has the potential to increase **integrated, coordinated**, and **open science**. During project inception and development, researchers could build in ways to involve stakeholders and the public, ranging from defining the scope and priorities of research questions and applications with community expertise to engaging the public in community science data collection (**networked**). The requirement that publicly funded research projects in New Zealand directly involve the native Māori people in project design, execution, and communication of findings has shown that such practices ensure measurable outcomes to research and society (Ministry of Research, Science & Technology, 2005). Increasing research team diversity by involving community members, stakeholders, and other non‐professional scientists can amplify data collection by orders of magnitude beyond what researchers alone can achieve and can increase the extent to which science is integrated, open, coordinated, and networked. For example, the “Indigenous Symposium on Water Research Education, and Engagement,” held in Montana in 2018 (Chief et al., 2019) brought 36 indigenous scientists, community activists, and elders together to discuss topics ranging from groundwater contamination to climate change, topics that are impacting Confederated Salish and Kootenai Tribes. Representation among different genders, backgrounds, nationalities, and career stages expands perspectives in a project (Sandbrook et al., 2019). Local non‐scientists can be great assets to projects, bringing valuable contextual information (Roman et al., 2021). The American Geophysical Union's Thriving Earth Exchange offers one opportunity for scientists to connect with communities seeking science support to resolve challenges that require the expertise of scientists, such as issues associated with municipal water quality and community forest health. Existing community science projects such as those listed at scistarter.org can provide ready‐made infrastructure for engaging members of the public in data collection.

Alternatively, researchers may create their own community science project leveraging existing infrastructure like that housed at citsci.org and anecdata.org. Including social scientists on project‐teams can maximize societal benefits and use of project outputs by non‐scientist audiences (Enquist et al., 2017). Media coverage of research can result in a broader appreciation of research findings and the return on invested funds by the public. Science translation and communication can extend beyond the traditional news media and can be led by researchers themselves, using traditional outlets such as newspaper, radio, and television, in addition to social media (e.g., Twitter, Reddit, Facebook), blog posts, podcasts, and even comics (Pourret et al., 2020). Funding agencies and publishers could encourage or even require such science translation. This could take the form of non‐technical abstracts and reports published alongside research papers. Communication in multiple languages is crucial for the effective dissemination of scientific ideas (Márquez & Porras, 2020). Finally, annual and tenure reviews should incentivize researcher participation by acknowledging, funding, and rewarding the effort that community and stakeholder engagement and science communication efforts necessitate.

## 4 Field, Experimental, Remote Sensing, and Real‐Time Data for Biogeosciences

### 4.1 Current State of ICON

Biogeoscience research is often limited by observational and analytical constraints, and by the integration of concepts and applications across subdisciplines (e.g., land‐ocean fluxes in Kothawala et al., 2020). Recently, a proliferation of data networks and observatories have employed principles of ICON science to mitigate these challenges across scientific research: from data collection to publication. Well‐established field sampling networks and observatories, like NEON, ICOS, FLUXNET, and LTERs, generate coordinated data products across disparate study sites and consolidate them (e.g., https://lter.github.io/som-website/; Wieder et al., 2021). ICON principles are likewise evident in the findable, accessible, interoperable, and reusable (FAIR) data policies required by many journals, which require the provision of direct measurements from independent and less intensively sampled campaigns to public repositories (e.g., Environmental Data Initiative), thereby allowing for post‐hoc cross‐scale syntheses. These have increased the number of open‐access direct measurements across field, experimental, remote sensing, and real‐time data that have facilitated parameter‐specific database creation and “bottom‐up” scaling efforts. The growing availability of field‐deployable sensors enables real‐time data collection of biogeochemical processes and drivers that capture rare phenomena and short‐term processes that are critical to ecological monitoring, experimental studies, and predictive models. Openly real‐time data are increasingly available in networks like Next Generation Water Observing System (NGWOS), and ecosystem‐scale experiments such as Spruce and Peatland Responses Under Changing Environments (Krassovski et al., 2018) and Biosphere 2 Landscape Evolution Observatory (Volkmann et al., 2018). Additionally, there are coordinated projects to collect field and sensor data at a network of field sites (e.g., Drought‐Net, NutNet; Chabbi & Loescher, 2017) or under the same research infrastructure (e.g., AnaEE; Clobert et al., 2018).

Satellite remote‐sensing is another Earth system monitoring technology that has increasingly employed ICON principles. Efforts to openly disseminate remote‐sensing data have grown rapidly, from the opening of Landsat archives and the establishment of data processing and distribution centers like the Land Processes Distributed Archive Center, to the development of user‐friendly web portals like Earthdata Search and EarthExplorer. Increased coordination has enabled international orchestration of upcoming missions, as well as integrated data products like the Harmonized Landsat‐Sentinel data set, which combines the National Aeronautics and Space Administration (NASA) and the European Space Agency (ESA) satellite data. At the application level, user‐driven repositories like GitHub have enabled open data and code sharing, while cloud‐based platforms like Google Earth Engine have made large data sets, complex algorithms, and cloud computing networked and open.

### 4.2 Major Challenges

Interrelated issues of data availability, computational costs, monetary costs, time costs, researcher preferences, and data standards pose key challenges. For example, challenges exist in balancing geographic representativeness and the need for environmental–ecological stratification (Guerin et al., 2020). Geographic gaps are common in data networks, especially for emerging nations, which directly impact data integration and openness (Villarreal & Vargas, 2021). While satellite imagery and open‐access platforms for data acquisition and processing can partially mitigate these geographic biases, inequity in resources, training, and access due to political restrictions and low funding in emerging nations greatly limit seamless integration. Moreover, despite the potential for existing networks to provide networked research infrastructure for research (Hinckley et al., 2016), mission‐ and agency‐specific protocols can make integrating ICON principles across networks challenging. Research in biogeosciences is driven by exploration and hypotheses rather than by integration and networking alone. As such, ICON‐driven research ensures transparency and reproducibility, while advancing the investigation of large‐scale, context‐dependent biogeochemical questions. With the large‐scale questions that need to be addressed in biogeosciences today, overcoming the challenges that inhibit ICON principles will be essential.

### 4.3 Opportunities to Advance Biogeosciences Through Technology

Adopting ICON principles in biogeosciences provides many opportunities to expand our understanding of critical ecosystem processes. In particular, paired experimentation and field observations provide coordinated assessments across scales needed to resolve global biogeosciences challenges like ecosystem responses to climate change (Hanson & Walker, 2020; Hinckley et al., 2016). First, we advocate accelerating efforts to integrate multiple experiments at single sites and coordinate research efforts across networks to provide integrated data streams. For example, globally coordinated field campaigns and remote‐sensing data networks can advance the quantification of biogeochemical drivers and feedbacks across scales to improve continental assessments of emerging trends. Second, ICON principles should be more thoroughly embraced for real‐time data collection by expanding sensor availability, coordinating data standards and tools, and increasing open access. Especially important in this effort is the goal to increase sampling in underrepresented geographical areas and expand the reach of data networks to researchers in those regions. Third, to optimize these opportunities to incorporate ICON principles across and within all subdisciplines in the biogeosciences, there should be transparency in data, metadata, and methods in open publications (i.e., clear design descriptions, uncertainty estimates), and an effort to achieve standardization while allowing for site‐ and budget‐specific modifications when needed. Finally, the development of easy‐to‐use forecasting tools (e.g., web dashboards) for non‐specialist end‐users in conservation and ecological management should be prioritized. Advances in biogeosciences can then be more readily incorporated by practitioners, allowing them to overcome barriers to technology and information where the need is greatest, especially in underrepresented low‐ and middle‐income countries that are critical for expanding ICON science to the global scale.

## 5 Call for Action to Work Towards ICON Science

Great potential exists to better engage stakeholders, community members, and inclusive networks of global scientists in research efforts. We strongly encourage richer involvement with these audiences and more purposeful translation and communication of findings to society (e.g., Arora et al., 2019, 2021). ICON‐driven science will not only solve scientific gaps but also increase scientific equity, inclusion, and more fluid use of collective scientific knowledge. To implement ICON principles in biogeosciences, we call for a suite of actions on short‐ and long‐term horizons, focusing on a people‐centric approach toward capacity building, cultural shifts, breaking barriers through reduced entry costs, building research networks, and promoting community engagement with open and fair research practices. We also suggest developing interoperable methods and instrumentation to confront global challenges and solve key questions in biogeosciences.

## Data Availability

This research does not use any data or software.
